# Supplementary Motor Complex and Disturbed Motor Control – a Retrospective Clinical and Lesion Analysis of Patients after Anterior Cerebral Artery Stroke

**DOI:** 10.3389/fneur.2015.00209

**Published:** 2015-10-12

**Authors:** Florian Brugger, Marian Galovic, Bruno J. Weder, Georg Kägi

**Affiliations:** ^1^Klinik für Neurologie, Kantonsspital St. Gallen, St. Gallen, Switzerland; ^2^Sobell Department of Motor Neuroscience and Movement Disorders, University College London, London, UK; ^3^Support Center of Advanced Neuroimaging, Inselspital, Bern, Switzerland

**Keywords:** anterior cerebral artery, stroke, supplementary motor area, anterior cingulate cortex, Bereitschaftspotential

## Abstract

**Background:**

Both the supplementary motor complex (SMC), consisting of the supplementary motor area (SMA) proper, the pre-SMA, and the supplementary eye field, and the rostral cingulate cortex are supplied by the anterior cerebral artery (ACA) and are involved in higher motor control. The Bereitschaftspotential (BP) originates from the SMC and reflects cognitive preparation processes before volitional movements. ACA strokes may lead to impaired motor control in the absence of limb weakness and evoke an alien hand syndrome (AHS) in its extreme form.

**Aim:**

To characterize the clinical spectrum of disturbed motor control after ACA strokes, including signs attributable to AHS and to identify the underlying neuroanatomical correlates.

**Methods:**

A clinical assessment focusing on signs of disturbed motor control including intermanual conflict (i.e., bilateral hand movements directed at opposite purposes), lack of self-initiated movements, exaggerated grasping, motor perseverations, mirror movements, and gait apraxia was performed. Symptoms were grouped into (A) AHS-specific and (B) non-AHS-specific signs of upper limbs, and (C) gait apraxia. Lesion summation mapping was applied to the patients’ MRI or CT scans to reveal associated lesion patterns. The BP was recorded in two patients.

**Results:**

Ten patients with ACA strokes (nine unilateral, one bilateral; mean age: 74.2 years; median NIH-SS at admission: 13.0) were included in this case series. In the acute stage, all cases had marked difficulties to perform volitional hand movements, while movements in response to external stimuli were preserved. In the chronic stage (median follow-up: 83.5 days) initiation of voluntary movements improved, although all patients showed persistent signs of disturbed motor control. Impaired motor control is predominantly associated with damaged voxels within the SMC and the anterior and medial cingulate cortex, while lesions within the pre-SMA are specifically related to AHS. No BP was detected over the damaged hemisphere.

**Conclusion:**

ACA strokes involving the premotor cortices, particularly the pre-SMA, are associated with AHS-specific signs. In the acute phase, motor behavior is characterized by the inability to carry out self-initiated movements. Motor control deficits may persist to a variable degree beyond the acute phase. Alterations of the BP point to an underlying SMC dysfunction in AHS.

## Introduction

Voluntary and involuntary movements are generated and controlled by a complex bihemispheric neuronal network involving the primary motor (MI) and supplementary motor complex [SMC; consisting of the supplementary motor area (SMA) proper and pre-SMA], cingulate cortex, and dorsolateral prefrontal cortex as well as a number of subcortical brain structures such as the basal ganglia and the cerebellum. Motor areas supplied by the anterior cerebral artery (ACA) involve the SMC, the anterior and middle cingulate cortex, and the rostral section of the corpus callosum. This part of the motor network is particularly involved in the generation of self-initiated (i.e., volitional), complex movement sequences, inhibition of purposeless movements triggered by external stimuli such as the grasp reflex, error control during motor performance, and motor learning (1, 2). An electrophysiological measure of voluntary control is available with the so-called Bereitschaftspotential (BP). The BP is a negative potential over the vertex emerging approximately 1 s before the onset of a voluntary movement. The early component most presumably originates from the SMA, while the later component is mainly assigned to the primary motor cortex and the lateral premotor cortex (3). The BP probably reflects cognitive processes preceding the initiation of volitional movements (4). According to recent computational frameworks for action, both conscious awareness of intention and a sense of agency characterize voluntary movements (5). By applying direct electrical stimulation to the SMC, a conscious intention of moving can be provoked underlining its role in generating volitional movements (6).

As previously mentioned, ACA strokes lead to a severe disruption of the above-mentioned motor network. The clinical spectrum of disturbed motor control after ACA strokes may encompass signs such as involuntary grasping of nearby objects, utilization behavior, and intermanual conflicts (i.e., the two hands are directed at opposite purposes) with absence of volitional movements (7). Underutilization of one body side in the absence of relevant weakness or sensory disturbances or deficits of reflexes, as it can be observed in ACA strokes, has been summarized under the term “motor neglect” (8) Apart from limb weakness, the above-mentioned motor signs have been acknowledged as characteristic features of the so-called alien hand syndrome (AHS) (9, 10). Its first description was rendered by Goldstein in 1908 who reported “a type of apraxia with the feeling of estrangement between the patient and his hand” (11). In 1972, Brion and Jeydnak observed analogous symptoms in a patient with a corpus callosum tumor, which inspired them to coin the term “la main etrangère.” It was subsequently translated into the English term “alien hand” (12, 13).

It has turned out that the clinical picture of AHS is variable and reflects a spectrum of abnormalities in motor control rather than a homogeneous clinical entity (14). Dolado et al. proposed following hallmarks as essential for the diagnosis of an AHS: (i) a feeling of foreignness of the affected limb, (ii) failure to recognize ownership of it when visual clues are removed, (iii) autonomous motor activities that are perceived as involuntary and are different from other identifiable movement disorder, and (iv) attribution of an action to another subject due to lacking sense of agency (15). Lesions within the SMC, the cingulate cortex and the corpus callosum have often been implicated in the context of AHS (10).

Although AHS has been known for a very long time, there is no comprehensive clinical-anatomical correlation addressing impaired motor control in a larger number of ACA stroke patients. Hitherto, most of the published literature is restricted to case reports and case series [reviewed in (7, 16, 17)]. The only systematic approaches published suffer from methodological drawbacks, including definitions that are too wide apart with regard to disturbed motor control and/or the lack of using adequate imaging methods (16, 17). Therefore, the aim of this case series was to characterize the clinical spectrum of disrupted motor control, including signs attributable to AHS and to identify the main underlying neuroanatomical correlates (18). We hypothesized that an involvement of the SMC is essential for the occurrence of the AHS spectrum of disturbed motor control after ACA strokes.

## Patients and Methods

### Study population

Over a period of 6 years, patients with arm paresis or plegia, after circumscribed ACA infarction were identified at our center and included in this case series. Conscious awareness of intention and sense of agency of volitional movements of the affected limb, both thought to be key features of an AHS, were the main focus of this study (5). On the basis of these two key features, clinical signs of disturbed motor control were classified into three different groups (7, 10, 13, 15–17, 19–31). Group A included AHS-specific signs, namely (A.I) lack of self-initiated movements, (A.II) exaggerated (not suppressible) grasping and groping behavior, and (A.III) presence of an intermanual conflict (i.e., the two hands are directed at opposite purposes). Group B included clinical signs, which did not necessarily reflect disturbed awareness of intention and sense of agency. These symptoms were thus considered as non-AHS-specific signs of disturbed motor control: (B.I) maintaining a particular limb position after a preceding complex motor task (i.e., motor perseveration), (B.II) Co-activation of the contralateral limb during volitional movements of the ipsilateral limb (i.e., mirror movements), and (B.III) any form of tremor. Group C included symptoms, which were signs beyond disturbed motor control of the upper limbs: (C.I) in this group, gait apraxia was expected (21). The study was approved by the ethics committee of the Kanton St. Gallen and was conducted according to GCP guidelines.

### Epidemiological data and clinical tests

Demographics and disease characteristics, including National Institute of Health Stroke Scale (NIHSS)-scores and stroke etiology according to TOAST criteria (32), were taken from the patients’ records. All patients underwent a standard neurological examination. The following procedures were used to screen clinically for the aforementioned clinical signs of disturbed motor control: (A.I) *impaired self-initiated movements* were studied by observing volitional gestures and the interaction with the examiner during taking the history and the clinical assessment. Furthermore, patients were asked to voluntarily perform tasks such as virtual piano playing or typing on a keyboard. Testing of muscle strength was difficult in the acute phase due to the inability to perform voluntary movements, but weakness was excluded in the subacute stage in all cases. (A.II) *The presence of an intermanual conflict* was evaluated by antiphasic upper limb movements (i.e., windmill-like movements of both arms), transferring objects from one hand to the other or by performing bimanual tasks (e.g., putting on glasses). A marked shift or loss of phase, disturbances on performing coordinated bimanual tasks, and purposeless counteracting of upper limbs during bimanual tasks were attributed to the presence of an intermanual conflict. (A.III) *Exaggerated grasping behavior* was tested by moving objects nearby in the visual field and by asking them to suppress compulsive grasping. Patients were also observed when they released objects or when they transferred objects from one hand to the other. (B.I) *Motor perseveration* was defined as maintaining a particular hand position, which was clearly related to a preceding (complex) motor task. (B.II) *Mirror movements* were picked up during the assessment of the affected hand by observing the contralateral one and vice versa. (B.III) We also screened our patients for any form of resting, postural and action *tremor*. (C.I) *Gait apraxia* was assessed in those patients who were able to walk independently. They were asked to walk along the corridor and to turn toward and away from the affected side. Shuffling gait with high cadence and paroxysmal interruption of locomotion, with trembling of the feet in place and preserved (seemingly paradoxical) ability to increase step length and height when stepping over an object on the floor or when presenting cueing signals, were considered as signs of gait apraxia (33). Patients were asked if they had the feeling of their feet being glued on the floor.

Typical clinical signs of disturbed motor control in the context of an ACA stroke, as specified above, were documented according to a predefined protocol. In nine patients (with exception from patient P6), videos of the clinical examination as detailed above were available for retrospective review. In P6, who explicitly declined video monitoring, symptoms were documented in detail in his hospital files. Symptom severity and persistence were rated by a neurologist in a semi-quantitative manner: clinical symptoms were considered as severe (+++), if they were permanently present and/or if they were a relevant source of impairment in the patient’s ability to carry out the clinical test. Severity was considered as moderate (++), if symptoms were frequently present, but only mildly interfered with the patient’s ability to carry out the clinical test. If there was just a hint of a particular sign or if the respective sign occurred only rarely, it was considered as mild (+). Absence of a particular sign was rated as “0.” Notably, due to the lack of validated clinical scores, this scale has been designed for the purpose of this case series. To assess the reliability of this rating, a second blinded examiner rated the videos and the interrater reliability rate (IRR) was calculated by the means of kappa statistics. Calculations yielded a kappa coefficient of 0.83 ± 0.12 observed as proportion of maximum possible kappa thus indicating a good IRR.

### Bereitschaftspotential (readiness potential)

The BP was recorded by using an EEG-EMG polygraphy. The EEG electrodes were placed over C3, C4, and Cz and the reference over Fpz according to the 10–20 EEG system. The ground electrode was fixed at the ear lobe. Patients were asked to keep their eyes closed and to repetitively perform briskly initiated middle finger extensions of 1-s duration in a self-paced manner, with an interval between each movement of approximately 6–7 s. Before the actual recording, they were instructed how to perform the finger movements while getting the sense for timing and movement initiation. To generate entirely self-initiated movements, they were instructed not to count or to pace the movement onset by using any other form of rhythmical encoding (e.g., by humming). They were also advised to fully shift their attention on the finger movement and to avoid falling asleep. Muscle activity was recorded from the long finger extensors by surface EMG. To avoid blinking, particularly at the time of movement initiation, we positioned two small sand bags over their eyelids. Eighty to 100 sequences of middle finger extensions were recorded from each hand. The BP was calculated offline using the ASA software (ENT Enschede, Netherlands). At least 50 artifact-free EEG epochs lasting from 2.0 s before to 1.0 s after motor onset were chosen and averaged for each limb separately. The BP was baseline corrected by averaging the epoch 1.5–2.0 s before motor onset. The amplitude at 0.25, 0.50, 0.75, 1.00, 1.25, and 1.25 s before and 0.25, 0.50, 0.75, and 1.00 s after motor onset as well at motor onset was calculated by averaging all data points acquired 50 ms before and after each respective time point (34). The results were then plotted against the grand average of the BP from 13 healthy controls.

### Lesion summation mapping

Images were acquired within the first days after hospital submission (median 2.5 days; range 0–30). Isotropic diffusion weighted imaging (DWI) sequences and T1 sequences were acquired in a 1.5 T or a 3 T MRI scanner (T1: slice thickness 5 mm, DWI: b = 1000 s/m2, slice thickness 4 mm). We used DWI sequences for lesion analysis as they showed the best contrast for ischemic brain tissue. In two patients, only CT scans were available. In both patients, however, the scans already showed a clearly demarcated ischemic brain lesion. Hence, they were feasible for reliably drawing lesion maps and were included for further imaging analysis.

For pre-processing of the scans, DWI and T1 sequences were first co-registered using Statistical Parametric Mapping 8 (SPM8)^1^ (18, 35). According to the general agreement for working in the stereotaxic standard space, the anterior commissure was defined as the origin of the coordinate system in all scans (MNI-coordinates *x* = 0, *y* = 0, *z* = 0). The ischemic lesions were drawn manually on the DWI sequences using the freely available MRIcron software^2^ and the drawings were put together to a 3D volume of interest (VOI). Both DWI and T1-weighted sequences were normalized to a MRI template and T1-weighted images were segmented by the means of the Clinical Toolbox running on SPM8^3^. The lesion maps were entered as masks in the algorithm for cost function masking to avoid distortion of the voxels within the ischemic lesion during spatial normalization. CT normalization routine integrated in the Clinical Toolbox was used analogously to normalize CT scans to a standard space template. Afterwards, all lesions were flipped to the left side to enhance power of the analysis. In the patient with a bilateral ACA infarction, the larger hemispheric volume defect was accordingly flipped to the left side.

Calculation of lesion maps was done in three steps. (1) Weighted summation (overlap) maps were calculated in SPM 8 for each clinical sign. Only VOIs from patients showing a particular sign were included in the retrospective calculation (see Table 1). A Kernel filter with 4 mm full-width half maximum was used to slightly smooth the summation maps. Each map was then thresholded to voxels damaged in >25% of our patients showing the respective clinical sign. (2) Summation maps for each symptom group (A, B, C) were created by using the image calculator function integrated in SPM8. The respective summation maps were calculated by summing up the summation maps of the different symptoms included in groups A and B, respectively. The summation map of group C was identical to the map for gait apraxia and therefore did not require further calculation. (3) To address the question which part of the ischemic lesions contributes to disturbed motor control of upper limbs in general, the union set of group A and B (A∩B) and the set difference of (A∩B)∖C were calculated. To address the question which brain section is specific for symptoms of the AHS spectrum, the set difference of A∖(B∪C) was calculated. Prior to calculation of all these sets, each group summation map was transferred into binary maps using the SPM8 image calculator.

**Table 1:** **Baseline demographic data and clinical findings**.

No	Age (years)	Sex	First ever stroke	Stroke etiology	NIHSS-score
P1	83	Female	Total right-sided ACA stroke	Cardioembolism	15
P2	82	Male	Partial left-sided ACA	Cardioembolism	17
P3	74	Male	Partial left-sided ACA	Cardioembolism	21
P4	87	Female	Total right-sided ACA infarct	Cardioembolism	7
P5	63	Male	Total bilateral ACA infarct	Cardioembolism	15
P6	75	Male	Partial left-sided ACA infarct	Cardioembolism	16
P7	69	Male	Partial left-sided ACA infarct	Large artery arteriosclerosis	3
P8	70	Male	Total right-sided ACA infarct	Large artery arteriosclerosis	6
P9	65	Male	Partial left-sided ACA infarct	Stroke of other determined etiology	11
				(Secondary to aneurysma coiling)	7
P10	74	Male	Partial right-sided ACA infarct	Large artery arteriosclerosis	2

*Stroke etiology according to Toast criteria; ACA, anterior cerebral artery; NIHSS, National institute of health stroke scale*.

In a final step, each lesion map was plotted onto the automated anatomical label (AAL) atlas using MRIcron and the involved brain areas as well as the center of gravity were identified by the respective built-in function. As the AAL does not distinguish between the pre-SMA and SMA proper, ROIs with the anterior commissural line as the border between these two areas (1, 36) were manually drawn in MRIcron and were used to determine the number of damaged voxels encompassed by each subsection.

## Results

### Study populations

Information from 10 patients aged between 63 and 87 years (mean 74.2) were available. Among them were eight males and two females. Initial NIHSS ranged from 2 to 21 points (median 13.0). Seven patients were followed up from the acute stage and three patients were added after reviewing our stroke database and clinical notes from the last 2 years. All patients investigated in the acute stage had disturbed conscious awareness of intention and sense of agency. The first signs of recovery occurring within days were involuntary finger movements elicited by touching their palm. In one patient, information on these features were missing. Five patients had an ischemic lesion within the left hemispheric ACA territory, four within the right and one had large bilateral ACA infarctions. The lesion pattern ranged from circumscribed infarcts confined to the SMA to bilateral territorial infarcts within the ACA territory. According to the TOAST criteria (32), macroangiopathy was identified as a stroke etiology in 3/10 patients, cardioembolic events in 6/10, and arterial emboli secondary to aneurysm coiling in the ACA in 1/10. Detailed clinical and radiological information are summarized in Table 1.

### Signs of motor control

Initially, all cases had marked difficulties to perform volitional hand movements. In the acute setting, 9/10 patients presented with an apparently severe paresis or plegia of one or both upper extremities, respectively (five right-sided, three left-sided symptoms, and one bilateral symptoms). In the subacute and chronic stage for all patients, movement initiation improved but signs of disturbed motor control such as exaggerated grasping, and disturbing movements of the affected limb persisted to a variable extent. These data are summarized in Table 2 and relate to the last control after the ischemic stroke (median duration of follow-up: 83.5; range: 7–585 days). Lack of self-initiated movements was present in 8/10 patients, intermanual conflict in 10/10 patients, exaggerated grasping and groping behavior in 6/10 patients, motor perseveration in 8/10 patients, mirror movements in 7/9 patients, tremor in 5/10, and gait apraxia in 4/8 patients. In the patient with bilateral ACA infarcts, a reliable evaluation of mirror movements was not possible due to the severe impairment of initiating movements of both limbs.

**Table 2:** **Disturbed motor control**.

No	I. Primary presentation	II. Motor signs at follow-up						
	AHS	Impaired self-initiated movements	Grasping	Intermanual conflict	Motor perseveration	Mirror movements	Tremor	Gait apraxia
P1	++	+++	+++	+	0	++	na	++
P2	++	+	++	++	+	+	0	+
P3	++	+	++	++	+	+	+	+
P4	++	+	0	++	+	+	0	+
P5	+	++	+++	++	+	na	++	na
P6	++	(+)	0	+	+	+	0	0
P7	na	(+)	0	++	0	0	0	0
P8	++	++	++	++	+	+	++	+
P9	++	0	++	+	0	+	+	0
P10	+	0	0	+	+	+	0	0

*Primary presentation (scoring): AHS, ++; minor or transient AHS, +*.

*Motor signs at follow-up (scoring): severe presentation, +++; moderate presentation, ++; mild presentation, +; symptom not present, 0*.

*na, information not available*.

### Imaging

**Figure 1:**
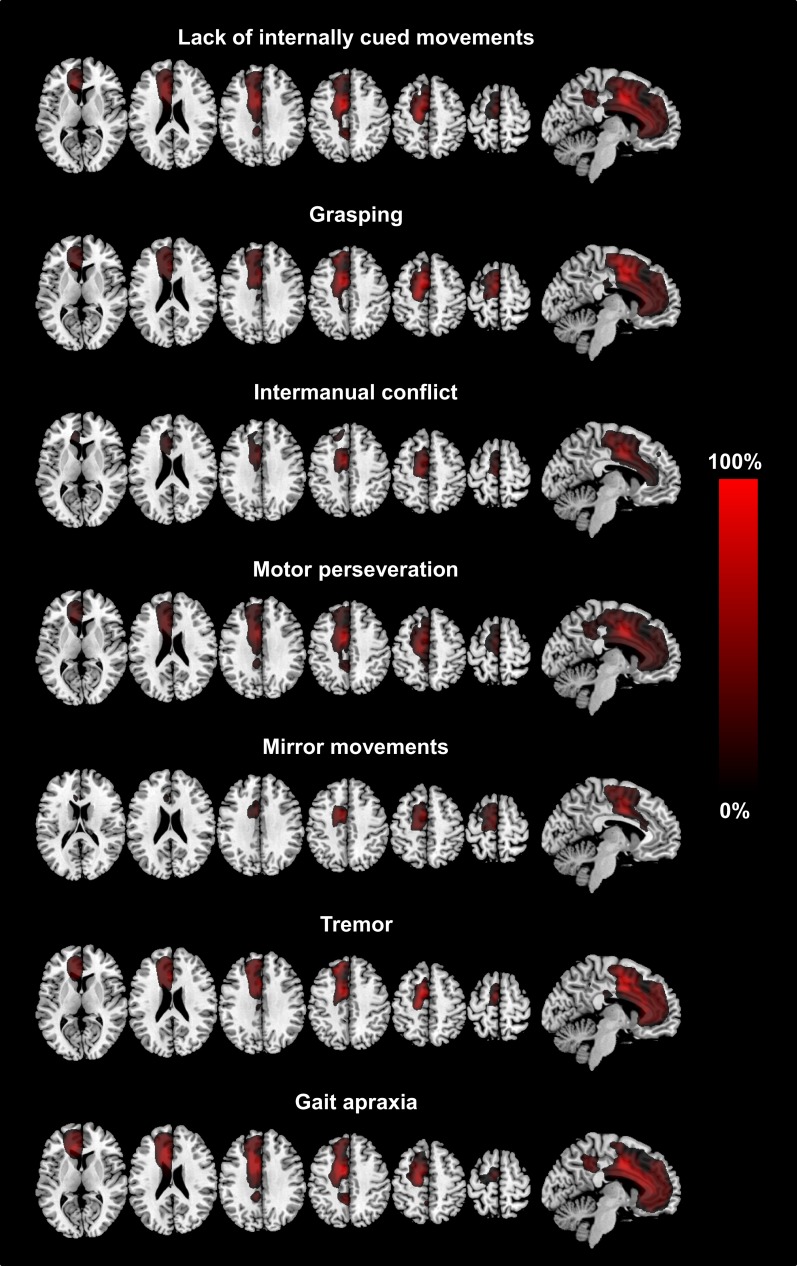
**The figure shows axial slices and a sagittal slice of a T1-standard MRI scan with the superimposed summation lesion maps for each clinical sign**. Each lesion map is thresholded at voxels damaged in >25% of patients showing the respective clinical sign. The legend (provided in percentages) refers to the total number of patients showing the respective clinical sign (MNI-coordinates: *z* = 8, 23, 33, 43, 53, 63 and *x* = −6, respectively).

**Figure 2:**
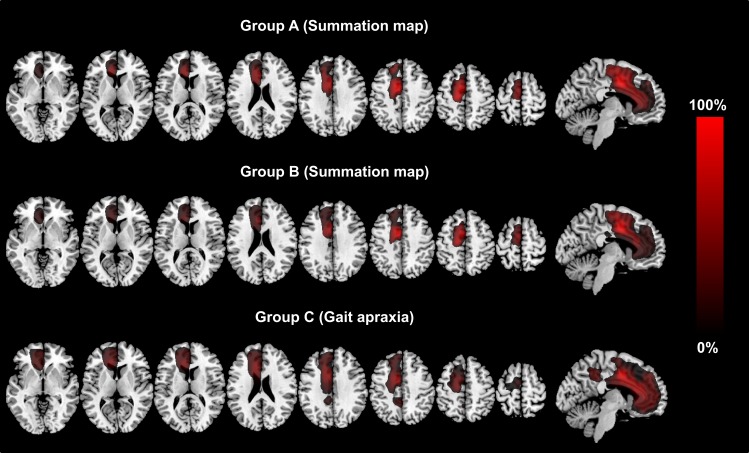
**The figure shows the lesion summation maps for group A, which included the lesion maps for all AHS-specific symptoms (grasping, intermanual conflict, impaired self-initiated movements). Group B encompasses all lesion maps for non-AHS-specific motor symptoms (motor perseveration, mirror movements, tremor). Group C (other signs) corresponds to the lesion map for gait apraxia**. The legend encodes the percentage of all patients*signs. The lesion maps are thresholded to voxels damaged in at least 25% of patients*signs and are plotted on axial and sagittal slices of standard T1 MRI scan (MNI-coordinates: *z* = −2, 8, 13, 23, 33, 43, 53, 63 and *x* = −6, respectively).

Table 3 summarizes the size of and the anatomical location of the weighted summation maps for each clinical sign. The total centers of gravity of the specific maps for grasping, intermanual conflict, lack of self-initiated movements, mirror movements, and motor perseveration were located in the caudal tier of the anterior cingulate cortex, whereas the center of gravity of the maps for gait apraxia and tremor were located within the white matter adjacent to the anterior cingulate cortex (MNI-coordinates: lack of self-initiated movements: *x* = −11, *y* = 9, *z* = 36; intermanual conflict *x* = −3, *y* = −3, *z* = 34; grasping: *x* = −10, *y* = 10, motor perseveration: *x* = −10, *y* = 10, *z* = 34; mirror movements: *x* = −7, *y* = 0, *z* = 42; *z* = 34; tremor: *x* = −3, *y* = −2, *z* = 27; gait apraxia: *x* = −13, *y* = 16, *z* = 27) (Figure 1). The combined summation map for all AHS-specific motor symptoms (i.e., group A) encompassed a total lesion volume of 99,863 voxels (corresponding to a lesion volume of 99.9 ml with a center of mass in the anterior cingulate cortex (*x* = −10, *y* = 12, *z* = 35). Similarly, the respective map for non-AHS-specific motor symptoms of the upper limbs (group B) had a lesion volume of 101,691 voxels (lesion volume: 101.7 ml) with its center of mass in the anterior cingulate cortex (*x* = −11, *y* = 11, *z* = 36) (Figure 2).

**Table 3:** **Anatomical details on the summation maps**.

	Total lesion volume					SMC							ACC					MCC					PCC					CC (genu)					CC body				
	Center of gravity			Volume		Center of gravity			Volume (SMAp)		Volume (pre-SMA)		Center of gravity			Volume		Center of gravity			Volume		Center of gravity			Volume		Center of gravity			Volume		Center of gravity			Volume	
	*x*	*y*	*z*	Voxels	%	*x*	*y*	*z*	Voxels	%	Voxels	%	*x*	*y*	*z*	Voxels	%	*x*	*y*	*z*	Voxels	%	*x*	*y*	*z*	Voxels	%	*x*	*y*	*z*	Voxels	%	*x*	*y*	*z*	Voxels	%
**AHS-specific**																																					
SIM	−13	16	27	104,842	100	−9	−1	47	3,577	3.4	6,845	6.5	−6	2	32	10,654	10.2	−8	1	43	11,384	10.9	−7	−40	34	463	0.4	−14	20	26	583	0.6	−14	−2	38	707	0.7
Grasping	−10	10	34	101,672	100	−10	−2	50	4036	4.0	7,852	7.5	−4	25	22	10,739	10.2	−9	−3	50	8,481	8.1	na	na	na	0	0.0	−4	28	12	583	0.6	−14	2	36	526	0.5
IMC	−3	−3	34	47,705	100	−10	−17	54	4,330	9.1	3,734	3.6	−5	8	32	4,270	4.1	−5	−3	40	6,962	6.6	na	na	na	0	0.0	−6	22	18	276	0.3	−14	2	36	387	0.4
**Non-specific AHS**																																					
Perseveration	−11	9	36	109,169	100	−6	1	47	3,910	3.6	6,864	6.5	−6	7	32	10,684	10.2	−7	−1	43	12,748	12.2	−7	−40	34	462	0.4	−14	20	26	583	0.6	−12	−4	36	708	0.7
MM	−7	0	42	35,929	100	−5	1	50	3,691	10.3	6,193	5.9	−3	14	32	1,166	1.1	−6	0	39	5,475	5.2	na	na	na	0	0.0	−6	24	16	20	0.0	−16	−2	38	119	0.1
Tremor	−10	10	34	81,362	100	−10	−2	50	3,026	3.7	4,834	4.6	−4	25	22	10,737	10.2	−9	−3	50	6,659	6.4	na	na	na	6,659	6.4	−4	28	12	583	0.6	−14	2	36	502	0.5
**Other signs**																																					
Gait apraxia	−3	−2	27	127,473	100	−9	−1	47	2,600	2.0	4,443	4.2	−7	5	32	10,920	10.4	−8	1	43	10,292	9.8	−7	−40	34	485	0.5	−14	20	26	583	0.6	−12	2	34	744	0.7

*Group analysis (N = 10). The table shows the location of the summation maps with regard to the involvement of various brain regions of interest. The proportion of the total lesion volume is shown for each brain regions (in voxels and in percentage of the total lesion volume). Furthermore, the center of gravity is provided for each brain regions. The coordinates (x,y,z) refer to the MNI standard space where all images have been normalized to. AHS, alien hand syndrome; ACC, anterior cingulate cortex; CC, corpus callosum; IMC, intermanual conflict; MCC, midcingulate cortex; na, not applicable; PCC, posterior cingulate cortex; SIM, self-initiated movements; SMA, supplementary motor area; SMAp, SMA proper; SMC, supplementary motor cortex*.

**Figure 3:**
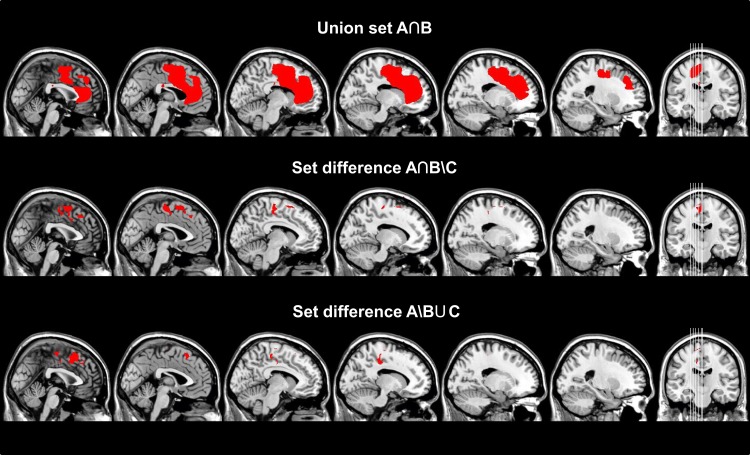
**Different sets (A∩B, A∩B∖C, A∖B∪C) calculated from the respective summation maps after transforming them to binary maps are superimposed on sagittal slices of a standard T1 MRI Scan (MNI-coordinates: *x* = −23, −18, −13, −8, −2, 0)**. The union set of group A and B (A∩B) and set difference (A∩B)∖C are thought to reflect the anatomical substrates for disturbed motor control in general regardless if the signs are specific for an AHS or not, whereas the set difference A∖(B∪C) reveals the anatomical substrates for AHS-specific symptoms. Interestingly, the latter set difference involves mainly the SMA, whereas disturbed motor control involves the SMC in addition to other regions of the frontal and rostral parietal lobe.

The union set of the maps for group A and B (A∩B) had a total size of 66,132 voxels (corresponding to a total lesion volume of 66.1 ml). 4.8% of the total lesion volume was located in the SMA proper, 8.5% in the pre-SMA, 14.4% in the anterior cingulate cortex, 10.0% in the MC, 6.5% in the genu, and 5.8% in the body of the corpus callosum. The remaining 50% of the lesion volume affected various other frontal brain regions. The calculation of the set difference of A∩B∖C revealed a total lesion volume of 9,394 voxels (total lesion volume: 9.4 ml). 8.1% of the lesion volume was found in the SMA proper, 25.2% in the pre-SMA, 13.1% in the midcingulate cortex, and 3.0% in the body of the corpus callosum. The set difference of A∖B ∪C encompassed 2,447 voxels (total lesion volume 2.4 ml). 0.6% of the lesion volume was located in the SMA proper, 32.3% in the pre-SMA, and 20.8% in the midcingulate cortex (Table 4; Figure 3). In between comparison showed that group A had the highest percentage of lesion load within the pre-SMA while the corpus callosum was not affected.

**Table 4:** **Percentage of lesion on regions of interest related to AHS associated and specific symptoms**.

	Total lesion volume		SMC				ACC		MCC		PCC		CC (genu)ww		CC (body)	
	Volume		Volume (SMAp)		Volume (pre-SMA)		Volume		Volume		Volume		Volume		Volume	
	Voxels	%	Voxels	%	Voxels	%	Voxels	%	Voxels	%	Voxels	%	Voxels	%	Voxels	%
A∩B	66,132	100	3,162	4.8	5,599	8.5	9,551	14.4	6,622	10.0	0	0.0	4,317	6.5	3,811	5.8
A∩B∖C	9,394	100	763	8.1	2,364	25.2	0	0.0	1,232	13.1	0	0.0	0	0.0	279	3.0
A∖(B∪C)	2,447	100	14	0.6	791	32.3	0	0.0	505	20.6	0	0.0	0	0.0	0	0.0

*The table shows the location of the intersections with regard to the involvement of various brain regions of interest. The proportion of the total lesion volume is shown for each brain regions (in voxels and in percentage of the total lesion volume). A, symptom group A; AHS, alien hand syndrome; ACC, anterior cingulate cortex; B, symptom group B; C, symptom group C; CC, corpus callosum; MCC, midcingulate cortex; na, not applicable; PCC posterior cingulate cortex; SMA, supplementary motor area; SMAp, SMA proper; SMC, supplementary motor cortex*.

### The bereitschaftspotential (readiness potential)

**Figure 4:**
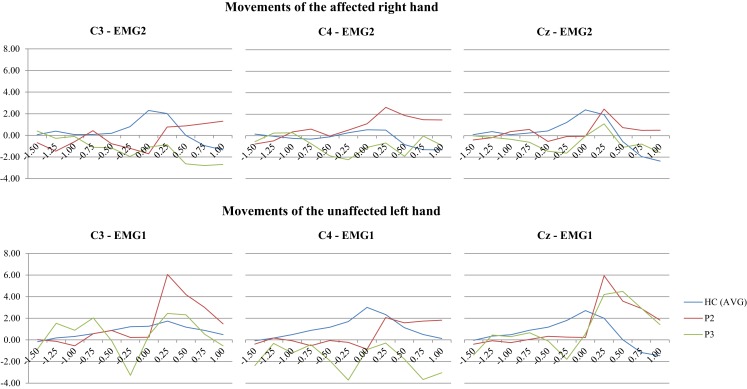
**EEG segments 1.5 s before to 1.0 s after onset of EMG activity in the finger extensors related to a briskly initiated extension of the middle finger were averaged (EMG 1: right finger extensors; EMG 2: left finger extensor)**. In contrast to healthy controls, the Bereitschaftspotential in the two patients with left-sided ACA infarcts does not show any negative shift over the hemisphere contralateral to the moved hand (C3). Interestingly, the BP was also attenuated over the unaffected right hemisphere (C4), when patients performed hand movements with their unaffected left hand. The results are plotted against a grand average of BP recordings from 13 healthy controls. Time is plotted on the *x*-axis (in second) and the BP amplitude on the *y*-axis (in μV). The nomenclature of the electrodes refers to the 10–20 EEG system.

The BP was recorded in two patients (P2 and P3). Both of them had a left-sided ACA infarct involving a large section of the vascular territory. Accordingly, the BP could not be detected over the contralateral hemisphere (corresponding to the electrodes C3) while performing finger movements with the affected right hand. Interestingly, a BP could not be recorded over the right hemisphere either (C4), when they performed the same task with the clinically unaffected left hand. The patients’ recordings are shown in Figure 4 (plotted against a grand average of BP recordings from 13 healthy controls).

### Illustrative cases reflecting the spectrum of disturbed motor control in ACA strokes

**Figure 5:**
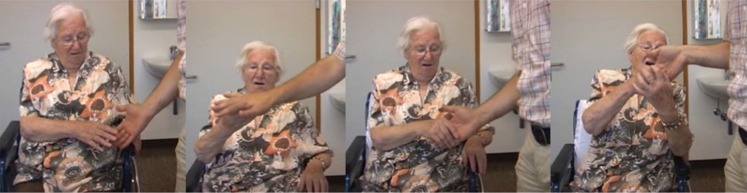
**The series of images illustrates the compulsive grasping behavior in P2**. The patient was asked to avoid grasping the examiner’s hand. However, the patient could not inhibit grasping. After she had taken the examiner’s hand, she could not release it without support by her left hand.

Of all cases, P1 (female, 83 years) with an ischemic lesion of the entire ACA territory, including the genu corpus callosum showed the most severe form of an intermanual conflict and exaggerated grasping. In the subacute stage, she was unable to perform bimanual tasks, e.g., putting on her glasses, as the affected limb counteracted the unaffected one. Moving objects in the nearby visual field led to compulsive grasping (magnetic hand) (Figure 5). After discharge from the hospital, she deteriorated progressively due to her deficient awareness of motor intention and self-agency. Therefore, she became severely impaired in managing her day-to-day life.

P2 (male, 82 years) with a large stroke in the left ACA territory did not show any volitional movements of the left upper limb on clinical testing in the acute phase. However, when he was explicitly asked to grasp the examiner’s hand, he was able to develop full strengths in keeping with a severe motor neglect. He also suffered from mildly exaggerated grasping. Intriguingly, he was not aware of preserved motor function and strength of the upper limb before. At the follow-up 5 months later, apart from a slight intermanual conflict, the motor neglect had resolved and motor control of the affected limb had recovered almost completely. He was fully independent in his daily life.

P5 (male, aged 63 years) experienced bilateral ACA infarcts and accordingly showed the most severe clinical syndrome of all our cases. The onset of his neurological symptoms and clinical course during the first 3 months after admission remain unclear, as he was found after lying on the floor for 1 day and signs of disturbed motor control were not sufficiently documented by the referring hospital. We saw him 3 months after stroke onset where he presented with akinetic mutism, an almost complete inability to perform volitional hand movement despite preserved limb strength, abasia, severely disturbed initiation of volitional movements, exaggerated grasping, motor perseveration, and mild bilateral action tremor.

Of all cases, P7 (male, 69 years) showed the mildest symptoms. He experienced a sudden feeling of a not obeying hand on the right side. These symptoms persisted for a few hours, but quickly improved thereafter. In the subacute stage, he had markedly improved but still showed a slight intermanual conflict and had slight problems with writing. The MRI revealed a small ischemic lesion within the left SMA.

## Discussion

In our case series, which is one of the largest imaging-based studies in AHS patients, we found a wide spectrum of clinical signs related to disturbed motor control ranging from very mild presentations with only transient impairment to very severely affected cases. Some of these symptoms, which we observed in our patients, have been well described in previous papers on AHS (7, 10, 13, 15–17, 19–31), while features such as mirror movements, motor perseveration, or the occurrence of an action tremor have so far not been reported in association with ACA strokes. In this case series, we were confronted with the interesting phenomenon of an initially apparent paresis/plegia of the upper extremity with significant recovery of muscle strength over the subsequent few weeks. The recovery curve of muscle strength, however, was more rapid and more favorable than it would have been expected in patients with weakness due to a corticospinal tract lesion. This observation points to alternative explanations of impaired upper limb function such as a severe motor neglect in the acute stage. The first signs of motor recovery in our patients were movements triggered by external stimuli such as touching their hand. Interestingly, patients were neither aware of the underlying motor intention nor of the self-agency of their movements, thus suggesting a full-blown motor form of AHS (15, 20, 24). The dissociation between self-initiated and externally triggered movements is essential, because they are largely dependent on the medial motor system supplied by the ACA, whereas the latter mainly rely on the lateral premotor system (supplied by the MCA) (37). A few patients presented with mild or only transient signs of AHS, as reflected by disturbed motor awareness in the acute, but not in the chronic phase. However, at the follow-up they still showed some signs of disturbed motor control as seen in the more severely affected cases, though to a much milder degree. This underscores the notion that the presentation of an AHS has a wide clinical spectrum.

Moreover, we were interested whether clinical signs of AHS (as defined as lack of conscious awareness of intention and the sense of agency) and non-AHS-specific signs, commonly observed in association with AHS, are caused by different lesion patterns. We could demonstrate that both AHS-specific and non-AHS-specific signs trace back to lesions within the SMC, and the anterior and medial cingulate cortex. This result was not entirely unexpected due to the important role of these brain areas in voluntary motor control (i.e., self-initiated movements and suppression of externally triggered motor subroutines) (1, 2). Our results are in line with a previously published retrospective analysis of 100 ACA strokes, which showed that motor disturbances were by far the most common signs (17).

A main finding of the present study is the predominant involvement of the pre-SMA in AHS-specific signs as shown by the approach with different set differences. This is a novel finding for ACA infarcts, but consistent with results from fMRI studies in healthy persons showing greater activations in rostral parts of the SMA after self-initiated movements (37). The pre-SMA, projects both to the lateral premotor cortex and the caudal parts of the SMA (38), although latter is not considered to play a major role in movement preparation as its projections descend directly through the pyramidal tract (39, 40). Gait apraxia was selected as a “reference”-clinical sign not associated with AHS and not affecting the upper extremities, but known to occur in lesions involving the medial frontal lobe. In line with this, gait apraxia was associated with lesions affecting the cingulate cortex in our study (21).

Previous studies of clinical-anatomical correlation in ACA stroke patients were biased mostly because of the approach with semi-quantitative analyses of predefined regions of interest. In the work of Chang and colleagues, AHS was associated with a combined involvement of the medial frontal lobe and the corpus callosum. An isolated or predominant affection of the cingulate cortex was found to result in an intermanual conflict, while medial frontal lesions were more likely to present with grasping behavior (10, 16). More recently, Sarva and colleagues published a systematic review of the literature on AHS (7). They concordantly found that the SMC, cingulate cortex and corpus callosum were the most commonly affected structures in the “*frontal*” AHS variant. Predominant involvement of hemispheric structures more frequently led to involuntary grasping and groping behavior, whereas an intermanual conflict was the most frequent clinical sign in callosal lesions. Our findings, however, do not favor the same relevance of the corpus callosum for clinical signs of AHS as suggested by these authors.

There were some clinical signs, which have not yet been described as common signs in ACA strokes. Mirror movements are usually seen in early childhood due to mutations in the DCC and RAD51 genes (41), although they may sometimes also occur in patients with basal ganglia disorders and strokes, mainly of the corona radiata. However, they have rarely been described in association with ACA strokes (26, 42). Functional MRI revealed that mirror movements are paralleled by bilateral activation of M1 and the SMA (25). Mirror movements probably occur due to an insufficient interhemispheric inhibition of the motor cortex located ipsilateral to the moved limb by a network, which connects the SMC, dorsolateral PFC, and M1 (43). The unilateral tremor of the affected hand we observed in some patients also deserves further consideration. It occurred in all patients as a new clinical sign with a latency of a few weeks after stroke. To our best knowledge, there are hardly any comparable reports of a hand tremor associated with ACA ischemia in the literature (25, 31). Stroke-associated tremor has mainly been reported in lesions of the thalamus, and the striatonigral, cerebello-thalamic or dentatorubrothalamic pathways (44). Clinically, the observed tremor resembles that of a dystonic tremor with a strong tendency to occur during action (45). In line with one previous report, the tremor was mostly seen just transiently (31). An association of the tremor with SMA and cingulate cortex lesions is of interest because an abnormal overactivity of these brain regions was found in an fMRI study in essential tremor (46). Our observations may thus suggest that an impaired function of the SMC or cingulate cortex may play an important role in the generation or suppression of pathological oscillatory network activity.

Our findings underpin the crucial role of lesions involving the SMC and cingulate cortex for disturbed motor control after ACA infarcts. Error detection and conflict monitoring have previously been attributed to the anterior cingulate cortex (2). The SMC, in turn, is more important for the generation of self-initiated movements, generation of complex motor tasks and the suppression of stimulus-driven, though, purposeless movements (i.e., grasping) (1). In this context, the BP is also of interest since it presumably originates from the SMC and reflects cognitive motor control prior to voluntary movements (3). So far, there are only two case reports of ACA strokes, which included BP recordings. In line with our findings, the BP following movements of the affected hand was also attenuated there (28–30, 47). Notably, the BP was also attenuated in our patients when they performed finger movements with their non-affected hand. This might indicate disturbed interhemispheric activation following a unilateral SMC lesion.

This analysis has several limitations. Due to its design as in parts retrospective case series, follow-ups were not standardized and patients were seen at different latencies after their strokes. Therefore, transient neurological signs may have been missed. Furthermore, this lack of standardized time intervals between the assessment in the acute stage and the follow-up assessments does not allow drawing definite conclusions to the clinical and functional outcome of these patients. A limitation of our clinical approach is the fact that it has not been validated elsewhere and there are no validated clinical scores for AHS symptoms in the literature. Therefore, we invented a semi-quantitative rating for our case series, which was proven here to have a very good IRR. Furthermore, the fact that just two patients underwent BP recordings does not allow to draw final conclusions on the BP in ACA strokes, since this potential is quite variable despite optimal recording settings (28). We acknowledge that a larger number of patients would also have increased the statistical power here. Moreover, our imaging results may have been flawed because of the different imaging methods used. A CT scan yields a different image of the brain in terms of contrast and distortion as an MRI scan. We attempted to overcome this concern making use of validated CT and MRI templates for spatial normalization (43). Nonetheless, we decided to include the two ACA stroke patients with CT scans, because otherwise we would have abolished two clinically very interesting patients from our series. A theoretical issue is the definition of an adequate threshold to delineate damaged brain voxels for lesion analysis. In order to avoid false negative results in this small patient cohort, we went for a rather low threshold of more than 25% of individuals to explore the defined motor signs by voxel-based symptom lesion mapping (35). Furthermore, we determined lesion location as the basis of anatomical probability atlases, which do not account for individual variability of neuroanatomy.

## Conclusion

In summary, AHS is primarily characterized by disturbed conscious awareness of intention and a deficient sense of agency for voluntary hand movement. This is reflected by the inability to carry out self-initiated movements while externally cued movements are neither suppressed nor perceived by the subjects. Common motor signs following ACA strokes are disturbed self-initiated movements, grasping and groping behavior, intermanual conflict, motor perseveration, mirror movements, and coarse tremor. Their occurrence is mainly associated with lesions of SMC, as well as the anterior and midcingulate cortex. The motor signs specifically related to AHS, i.e., disturbed self-initiated movements, grasping and intermanual conflict, are mainly related to lesions of the pre-SMA and MCC. To date, little is known about the clinical course and long-term outcome of these patients or about the best approach on how to rehabilitate these patients. Therefore, further studies in these patients are warranted.

## Conflict of Interest Statement

The authors declare that the research was conducted in the absence of any commercial or financial relationships that could be construed as a potential conflict of interest.

## Footnotes

^1^http://www.fil.ion.ucl.ac.uk/

^2^http://www.mccauslandcenter.sc.edu

^3^http://www.mricro.com/clinical-toolbox/

## Abbreviations

ACA, anterior cerebral artery; AHS, alien hand syndrome; BP, Bereitschaftspotential; MNI, Montreal National Institute; NIHSS, National Institute of Health Stroke Scale; SMA, supplementary motor area; SMC, supplementary motor cortex; SPM, statistical parametric mapping; VOI, volume of interest.
